# Mainstreaming gender and promoting intersectionality in Papua New Guinea’s health policy: a triangulated analysis applying data-mining and content analytic techniques

**DOI:** 10.1186/s12939-017-0555-5

**Published:** 2017-04-20

**Authors:** G. Lamprell, J. Braithwaite

**Affiliations:** Centre for Healthcare Resilience and Implementation Science, Australian Institute of Health Innovation, Macquarie University, Level 6, 75 Talavera Rd, Macquarie, NSW 2109 Australia

**Keywords:** Health policy, Health services research, Health equity

## Abstract

**Background:**

Gender mainstreaming is an approach to policy and planning that emphasizes equality between the sexes. It is the stated policy for gender equity in Papua New Guinea’s (PNG) health sector, as well as all other sectors, and is enshrined in the policies of its biggest aid givers. However, there is criticism that gender mainstreaming’s application has too often been technocratic and lacking in conceptual clarity not only in PNG but elsewhere. In the health sector this is further exacerbated by a traditional bio-medical approach, which is often paternalistic and insufficiently patient- and family-centered.

**Methods:**

This study analyses the policy attitudes toward gender in PNG’s health sector using both data-mining and a traditional, summative content analysis.

**Results:**

Our results show that gender is rarely mentioned. When it is, it is most often mentioned in relation to programs such as maternity and childcare for women, and elsewhere is applied technocratically.

**Conclusion:**

For PNG to promote greater levels of equity, the focus should first be on conceptualizing gender in a way that is meaningful for Papuans, taking into account the diversity of experiences and setting. Second, there should be greater focus on activists and civil society groups as the stakeholders most likely to make a difference in gender equity.

## Background

### PNG: Key challenges

In PNG women and children suffer some of the worst health outcomes in the world. One in every 13 children dies before they turn five [1]. Maternal mortality rates are amongst the highest anywhere, and the highest in the Pacific at 230 deaths per 100,000 live births [2]. The risks for maternal deaths have increased due to a combination of high fertility levels and lack of skilled health personnel, who attend only an estimated 53% of births [3].

For both women and men the major causes of morbidity and mortality are communicable diseases such as malaria, tuberculosis, diarrheal diseases, and acute respiratory disease [1]. HIV/AIDS is also highly prevalent, affecting nearly 1% of the adult population [4] and in 2013 a reported 0.94% of pregnant women attending antenatal clinics had HIV [3]. There are signs of progress with declining malaria rates and improvements in care and treatment of HIV/AIDS, which recently moved from classification as a generalized epidemic to a concentrated epidemic [5, 6]. However, overall, the situation remains dire and is deteriorating on many other indicators [7].

Progress in the delivery of better health care in PNG is significantly limited by multiple, interrelated factors. The most basic are related to resources and access. Over 800 indigenous languages are spoken within many traditional societies spread across 22 provinces [4]. The majority of the population, some 87%, lives in rural areas, occupying diverse islands and rugged terrain [8]. The geographic isolation of the many rural communities affects both the availability of health workers and the delivery of supplies, and inhibits the spread of new ideas and sound practices, adding considerable obstacles to accessing quality services [9]. The percent of GDP expenditure on health care is low by international comparison [10] and what funds there are have been vulnerable to systemic corruption [11].

### Socio-economic and socio-cultural factors

Funding and delivery of, and access to, health care for any Papuan is challenging. However, it is socio-economic and socio-cultural issues that are at the core of women’s health care in PNG [12].

The subordination of women in PNG society and politics is systemic and alongside efforts, forced or otherwise, at modernization, there is evidence this situation is becoming more fiercely enacted [13]. Efforts to move forward with women rights often clash with traditional beliefs. Sorcery-related violence is a problem [11, 14]. With “widespread and systemic patterns of abuse perpetuated by police and endemic violence against women and children by male relatives and both known and unknown perpetrators” [15], violence and sexual abuse are among the most common factors affecting women’s poor health status. Human Rights Watch estimates that 17% of Papuan women will experience rape or sexual assault in their lifetime [11]. The threat of violence, amongst other things, perpetuates subversive forms of gender-based violence, affecting a women’s freedom of movement and ability to access resources [13]. Women are commonly denied access to justice or reparation and do not get to see the punishment of the perpetrator. Despite the introduction of a Family Protection Act in 2013, which civil society groups believed would help curb domestic violence, it has still not been implemented [11].

Gender equity policy must be available, strong and reinforced widely to contend with this adversity. The continuing poor health results for women, ongoing corruption across society and lack of political will toward the goal of gender equity suggest gender mainstreaming has failed in its goals, including in the health sector. Any political will–which seems to be merely lip service across PNG society–has not translated into the culture or behavior of either the citizens or the political parties who represent them and who create these policies. PNG women continue to suffer poor health status, disadvantage and discrimination [16].

### Gender mainstreaming as a failed approach?

Gender mainstreaming, or the incorporation of equity considerations into policies, programs, and regulations, has been proposed multiple times over the last 15 years as a solution, but it has not passed muster in PNG. There is criticism that gender mainstreaming’s attempts have too often been feeble and superficial, and when equality has been espoused, the application has been technocratic and lacking in conceptual clarity, not only in PNG but elsewhere [17]. Overall there is doubt as to its usefulness and ability to address the diversity of women and men’s experiences [18–20]. For Papuans, whose lives are ethno–linguistically exceptionally diverse, spreading from the urban centers to some of the most remote, rural settings in the world, with a huge variance in local traditions and language–and for Papuan women whose lives exist at the intersection of not just their poor socio-economic status but at the intersection of Papuan culture, tradition and history–attention to such diversity within policy is both beneficial and necessary. Yet progress has been, at best, painfully slow.

Beyond PNG, health sectors have long faced criticism for taking a myopically bio-medical approach to gender equity–one in which only the biological differences of women and men are considered. Studies have looked at determinants of health beyond the biological to show how gender and health are interwoven in complex ways in women’s lives [21–23]. The need to go beyond biological differences to an understanding of gender as a relational concept has also been recognized by feminist communities and a new wave of identity politics has emerged that recognizes gender and health as complex, crosscutting issues. Despite this, in both the developed and developing worlds, policy and programs are still not effectively addressing the challenges [24]. Whilst gender mainstreaming does not preclude stand-alone, sex-specific interventions, these are both necessary and beneficial to combat inequality, the formation of such policies such as those directed at maternal health or infant mortality rates is often seen as sufficient in itself [25]. Gender mainstreaming will have little effect as an emancipatory project if applied technocratically, episodically and unsustainably, and where it fails to take account of the crosscutting variables it needs to effect.

To make progress, we must understand what policy is trying to do and how it might be strengthened. The continued poor health status of PNG women, criticism of gender mainstreaming in its technocratic application, conceptual ambiguity and inability to deal with complexity or counter-balance the dominant bio-medical approach, are all factors which need to be assessed. The growing call for a more complex notion of self in feminist thought and identity politics is also at issue here. It is highly relevant to study and analyze the policies that in theory should protect the lives and health of PNG women and their children, even with the complexity of their problems and their settings.

### Research principles and overarching approach

Here, we analyze the policy settings addressing gender in PNG’s health sector using text data-mining techniques and summative content analysis to get at the heart of what is being espoused in the policy landscape of PNG. Text data - mining examines key words, concepts and themes in documents, derives the most salient contents especially of large documents, and creates a visual map of conceptual and thematic connections. When people read texts, they import their own prejudices, understandings and idiosyncrasies. Textual data mining is less likely to be biased, as it cannot be dictated by the preconceptions of the researcher. Its use in this kind of analysis is uncommon, and although it has been used previously in studies of policy documents and guidelines [26–28], we can find no parallel study of gender analysis taking such an approach, and no study of developing countries and their policy considerations. This points to the timeliness of this research. We complement textual analysis with content analysis.

### AIM

The aim of the study (Aim 1) is to assess whether, the extent to which, and how gender has been represented in PNG’s health sector policy, and the themes and concepts as they sit embedded in policy documents and frameworks. Then (Aim 2), armed with this analysis, a set of recommendations for strengthening gender equity for the benefit of PNG’s health system are made.

## Methods

As indicated, two complementary analyses of the literature were undertaken. We sought triangulation of the methods.

### Text analysis using data-mining

We first applied data-mining techniques provided by Leximancer, an automated content analysis computer program. We used the most recent advances, encapsulated in version 3.0, to execute text mining of our chosen policy documents. Leximancer first examines word segments and builds a thesaurus of significant terms, called textual ‘concepts’ [29]. Second, Leximancer facilitates the examination of the connectivity and relationship between the concepts. The emergent groups of the most highly connected concepts are referred to as ‘themes’. Leximancer produces a ranked list of themes and concepts as well as a map of their connections, providing details both of the weight of a concept within the text and the strength of its connection to other concepts.

Leximancer is unique for a number of reasons. It goes beyond mere counting of words, by containing a sophisticated thesaurus, and allows deep interrogation of concepts and themes. It not only produces rankings of the most prominent concepts and themes but the visual mapping feature also provides insights into the relationship between them. The map facilitates the possibility of revealing associations that could otherwise be missed [30].

Policies were collected from the National Department of Health PNG website in early 2015. Policies that specifically dealt with gender, such as the National Health Sector Gender Policy (2014), were excluded as this was not our target research question, and doing so would likely skew the results. We ran the Leximancer analysis on eight core policy documents in total (Table 1). The included policies are representative of the policy landscape of PNG and are particularly pertinent to the aims of stakeholders to achieve a better society and health sector for PNG, along with fair, just and equitable health care and treatment in acute and community settings. A priori, at the heart of such policy lie plans for an effective delivery system modeled on others in both developed and developing countries, and relying on gender mainstreaming both for providers and patients.

**Table 1:** PNG policy documents for analysis

POLICY	DESCRIPTION	YEAR
*National Health Plan 2011–2020* [26,291 words]	This is the single governing policy for the health sector in PNG. It stipulates the areas for investment and policy directions for the health sector. The plan is in two volumes. Volume 1 includes the policies and strategies while Volume 2 provides background information for the development of the plan, and health and health services statistics. We ran Leximancer on Volume 1 only [1].	2010
*Free Primary Healthcare and Subsidized Specialized Service Policy* [4,571 words]	This policy sets out a system wide approach to fulfill part of an action plan set out by the government. It stipulates “The government will maintain its commitment to accessible and affordable health care by providing free primary health care and subsidized specialist services” [31].	2014
*National Policy on Health Promotion for Papua New Guinea* [8,436 words]	Sets out a health promotion program: “To empower individuals and communities thereby enabling them to control the status of their own health” [32].	2003
*PNG Community Health Post Policy* *[4,522 words]*	Provides direction to improve rural health service delivery [33].	2013
*National Research Agenda for HIV and AIDS 2008–2013* [7,023 words]	Describes research done to date and provides an agenda for future research [34].	2008
*The Development of PNG National Health + HIV Research Agenda 2013–2018* [24,241 words]	Describes the development of a strategic research agenda developed by the *Working Group for the development of the PNG National Health and HIV Research Agenda,* supported by WHO [35].	2013
*The National Drug Policy for Papua New Guinea* [5,480 words]	Sets out policies to promote healthy drug use and provide more affordable, accessible, safe and quality drugs [36].	1998
*Papua New Guinea Development Strategic Plan 2010–2030* [49,891 words]	Sets out a blueprint to achieve the targets and goals in PNG’s Vision 2050 policy [37].	2010

### Summative content analysis following text mining

The Leximancer analysis was supported by a more traditional, summative content analysis to explore how gender has been treated in the policies and ascertain whether gender has been well conceptualized. Has gender, for example, been treated mainly as a bio-medical construct, or applied technocratically? The summative content analysis was conducted by the first-listed author to assess and interpret the text [38], and the results reviewed by the second-listed author.

A researcher-conducted content analysis complements Leximancer as the latter gives us an overview of the major themes of the document whilst the former allows for a more in depth analysis and contextual understanding of the way in which gender has been considered in the documents. In this case, a traditional content analysis was particularly useful and necessary in order to appreciate nuanced use and meaning, allowing us to locate the terms in the text, record their frequency, find any meaningfully related terms, and to note the context of use.

The eight policy documents were each scanned and entered into a word-processing program to facilitate the researcher-conducted analysis. The total word count per document was determined through use of a computer word-count utility. We did not include tables, figures or supplementary material but did include title, main body of text, and summaries. There were a total of 130,255 words. The unit of analysis was specific key words related to gender. Analysis started with computer-assisted searches for occurrences of the terms “gender” or “gendered”, “women” or “woman”, “men” or “man”, “female” and “male”. Word frequency counts for each of the gender related terms were calculated and compared to the total word counts for all of the eight policies. Then, we read each word found in its context, seeking to understand how it was used and the circumstances of use.

## Results

### Leximancer

**Map 1:**
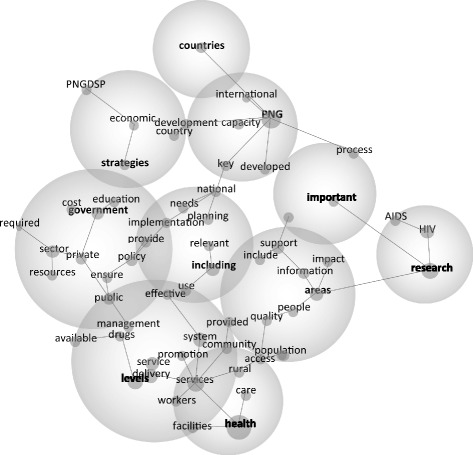
Concept map of policy literature. The map shows themes, i.e., groups of related concepts, represented by the circles. With Leximancer, concepts are denoted by dots, and the size of the concept dot is in direct relation to its significance compared with other concepts

The map shows themes, i.e., groups of related concepts, represented by the circles. With Leximancer, concepts are denoted by dots, and the size of the concept dot is in direct relation to its significance compared with other concepts (Map 1).

A total of ten themes emerged. These are listed in Table 2. Health is the largest theme encompassing the concepts of *services, care, rural, community* and *facilities*. The second largest theme, government, includes *sector, policy, public, provide, implementation, ensure* and *private.* There is a significant cluster around the concept of *services*, which is linked with *workers*, *community, promotion, delivery, rural* and *care*. These align with the expectation that PNG healthcare must target local level access and services. Including is a more technical theme and encompasses *implementation, national, use, effective, needs, planning* and *relevant. Research* is linked with *HIV* and *AIDS* in their own cloud, and with *areas* and *important* (Table 3).

**Table 2:** Themes in policy literature

Theme	Connectivity^a^
Health	100%
Government	88%
Levels	76%
PNG	62%
Areas	61%
Including	51%
Research	32%
Strategies	23%
Important	13%
Countries	03%

^a^The connectivity of concepts within the theme, relative to the most connected theme

**Table 3:** Concepts in policy literature. Concepts are divided as ‘Name-like’ or ‘Word-like’. ‘Name-like’ identifies proper names, e.g., PNG

Name-like	Count	Relevance^a^
PNG	811	62%
Government	321	25%
HIV	250	19%
PNGDSP	186	14%
AIDS	90	07%
Word-like	Count	Relevance
Health	1301	100%
Research	706	54%
Services	379	29%
Development	370	28%
Sector	348	27%
Levels	326	25%
Areas	297	23%
System	295	23%
Policy	250	19%
Including	236	18%
Service	233	18%
Delivery	216	17%
Capacity	206	16%
People	203	16%
Provide	194	15%
Implementation	191	15%
National	185	14%
Support	182	14%
Rural	181	14%
Quality	175	13%
Access	175	13%
Public	171	13%
Care	169	13%
Facilities	168	13%
Promotion	166	13%
Information	166	13%
Community	165	13%
Population	163	13%
Strategies	155	12%
Resources	155	12%
Important	152	12%
Education	149	11%
Ensure	144	11%
Use	144	11%
Economic	141	11%
Key	138	11%
Management	132	10%
Country	130	10%
Effective	129	10%
International	127	10%
Private	124	10%
Needs	119	09%
Available	117	09%
Required	114	09%
Impact	112	09%
Planning	105	08%
Process	104	08%
Based	102	08%
Developed	99	08%
Include	98	08%
Drugs	93	07%
Workers	92	07%
Provided	91	07%
Countries	91	07%
Cost	90	07%
Relevant	79	06%

^a^Frequency of segments of text coded with the concept, relative to the most frequently occurring concept

The Leximancer results provide us with a useful overview of the policy documents. Though the Leximancer software was able to give us words going down to just 6% relevance in the texts, no gendered terms were revealed, including *gender*, *women, men, female, male, girl, boy* or their plural forms, as themes, or concepts.

Whilst this makes it difficult to map these gendered terms with other policy elements, their absence is telling. We can see from the other concepts that a great deal of focus is on the technocratic operation of health services, including *process, facilities, cost, management and promotion*. This reflects the nature of the delivery of healthcare in PNG where infrastructure and access to services is lacking as the government and other groups struggle to stretch policy guidance and services across the difficult PNG terrain to the many rural communities. *Research* is both a significant theme and concept, reflective of the lack of reporting on effectiveness and outcomes of health programs and policy in PNG. However, focus on the technocratic and research aspects of the delivering of health care also perhaps denotes a lack of attention to specific areas of inequality. *HIV/AIDS* is the only target area revealed as a concept, reflecting the scale of the problem in PNG but also the level of donor attention to the issue.

### Researcher-conducted content analysis (Table 4)

**Table 4:** Frequency of keywords

POLICY	“gender” OR “gendered”	“Women” OR “woman”	“Men” OR “man”	“female”	“male”	TOTAL
*National Health Plan 2011–2020*	3	9	2	0	0	14
*Free Primary Healthcare and Subsidized Specialized Service Policy*	0	4	2	0	0	6
*National Policy on Health Promotion for Papua New Guinea*	1	1	0	0	0	2
*PNG Community Health Post Policy*	0	2	0	0	0	2
*National Research Agenda for HIV and AIDS 2008–2013*	17	10	6	0	0	33
*The Development of PNG National Health + HIV Research Agenda 2013–2018*	8	15	15	0	5	43
*The National Drug Policy for Papua New Guinea*	0	1	1	0	0	2
*Papua New Guinea Development Strategic Plan 2010–2030*	18	18	6	7	3	52
130,255 words in total	Frequency of keywords in texts					154

In three of the documents, the *Free Primary Healthcare and Subsidized Specialized Service Policy*, *PNG Community Health Post Policy*, and *The National Drug Policy for Papua New Guinea*, there is no instance of the word “gender” being used. The *Free Primary Healthcare and Subsidized Specialized Service Policy* mentions “men” in two instances and “women” in four. All are within the one page message from the minister for health and HIV/AIDS that preface the document [31].

The *PNG Community Health Post Policy* mentions “women” in two instances. The first is in discussion of the need to improve service delivery especially for “the most vulnerable members of our community - the women and children” [33]. The second is a bio-medical treatment of gender: “The input of women should be sought and prioritised at all stages to ensure their needs with respect to maternal and child health inform this process” [33]. *The National Drug Policy for Papua New Guinea* has just one instance of “man” and “woman”. Both appear in a one-line quote from the PNG Constitution [36].

The *National Policy on Health Promotion* uses “gender” in just one instance where it is listed among things like age and religion, in a two-sentence paragraph on health inequalities. “Women” occurs also in just one instance, to thank the Women and Children’s Health Project [32].

The *National Health Plan 2011–2020* refers to “gender” in just three instances. The first is the title of a ‘Vision 2050’ pillar of development ‘Human Capital Development, Gender, Youth and People Empowerment’ [1]. The second mention of “gender” is in relation to determinants of health: “Gender plays an important role in health choices and health outcomes, as well as age, with programs needing to target in accordance with sex and age-specific risks. More health information broken down by sex is essentially needed” [1]. The third mention is listed, again including age, religion and others, in relation to equity in health [1]. Maternal health is considered a ‘Key Result Area’ in the document; “women” appears nine times but almost all in relation to bio-medical issues. “Men” appears in two instances. “Male” and “female” appear once – both in relation to condom accessibility [1].

*The Development of PNG National Health + HIV Research Agenda 2013–2018* mentions “gender” in eight instances, “men” in fifteen instances and “women” in 15 instances. Six mentions of gender are in relation to “gender-based violence” [35]; one mention in relation to ‘HIV Strategic Research Priorities,’ which includes “Research on gender norms…” [35] and; the final mention is as part of a name title [35]. Eight of the occurrences of “men” and eight of the occurrences of “women” are in participant counts for workshops held [35]. “Men” also occurs twice in an example of disease-specific vulnerable populations “e.g., sex workers and men who have sex with men for HIV” and twice again when this example is later repeated [35]; it occurs again in ‘The Agenda’ from the workshops where they include “the role of men in maternal and child health” [35] The final two mentions of men are in the second part of the Annex under conclusions for Research Domain one “Maternal, reproductive and child health research”. Under both research on supervised delivery and family planning it is noted amongst other things “the role of men in this process is an important aspect” [35].

Of the seven other occurrences of “women” not used in relation to numbers of workshop participants, once is in a discussion of the need for greater inclusiveness of women at future workshops [35]; three times in the repeated “Research on the social implications of the establishment of industries, in particular extractive industries, and especially implications for women and children” [35]; and the final three occurrences in the summary of “Research on the prevalence, determinants and burden of violence…” [35]. “Male” also occurs in five instances, three related to circumcision, and two to male on male sex [35].

Two documents mention gender at a higher rate than the other policies, the *National Research Agenda for HIV and AIDS 2008–2013* and *Papua New Guinea Development Strategic Plan 2010–2030*. The *National Research Agenda for HIV and AIDS 2008–2013* refers to “gender” in 17 instances, “gendered” in one, “men” in six instances and “women” in ten instances [34]. “Gender” is first mentioned twice in the background for the paper, in relation to “gender-based violence” and “gender inequalities”. Two instances of “gender” are in relation to “gender-mainstreaming”; the first in a discussion of grant application processes; the second under HIV Best Practice Guidelines, which lists gender mainstreaming amongst other prevention efforts [34]. “Gender” is listed as a priority area for further study and occurs six times under this title, twice as “gender”, then as “gender-based violence”, “gender status”, and “gender roles” [34]. It is also mentioned as part of the priority area of “Illness and disease” under condom use and the need for “Understanding perceptions, influences on uptake, gender based negotiations, access and availability” [34]. “Gender” occurs three more times when these priority areas are later reiterated [34]. One mention is in naming of the ‘National Gender Policy and Plan on HIV and AIDS’, and the final two mentions occur in the references [34].

The first two mentions of “men” occur in the acronym description “MSM - Men who have sex with men” which is used twice in the document [34]. Three more mentions in the ‘Background’ to the paper, the first mentioning the inequality in access to education; the second as “a need to understand more about the lives of men and the role of power and masculinities” and; the third relating to risks of infection from transactional to both men and women [34]. The final mention occurs in the references. The first five mentions of “women” occur in the ‘Executive Summary’ and ‘Background’, in relation to gender-based violence, listed amongst vulnerable groups, or in relation to sexually transmitted disease [34]. One occurrence was listed as a key point for a research agenda workshop undertaken, ‘Women, Violence and Groups at Higher Risk’ [34]. The final four mentions are from the references.

The *Papua New Guinea Development Strategic Plan 2010–2030* mentions “gender” on 18 instances, “men” six instances, and “women” 18 instances. It also mentions “female” on seven instances and “male” on three instances [37]. One of the occurrences of “women” is as part of the acronym, ‘GDI - gender development index’. However, Part 6 of the document is dedicated to “cross-cutting” themes, one of which is ‘Gender’. All of the 17 other occurrences are within or related to the title of Part 6 section on ‘Gender’. The stated goal of this cross-cutting theme is “All citizens, irrespective of gender, will have equal opportunity to participate in and benefit from the development of the country” [37]. It lists key strategic areas including “gender empowerment”, “women and children as victims of domestic violence”, “Female to male enrolment rate”, “Females in tertiary education” and “Females in wage employment” [37]. It also gives strategies for enhancing gender equity. Whilst it mentions important and gendered areas for concern, it does not attempt to define “gender”, nor does it mention “mainstreaming”.

All instance of “men” and “women” occur under the ‘Gender’ section. However, women also occurs once as part of another cross-cutting theme, ‘Population’*; “*slowing population growth will occur by improving the education of women and girls, as well as boys”; and once as part of the acronym description ‘UNIFEM - United Nations Development Fund for Women’ [37]. “Female” occurs five times within the ‘Gender’ section but notably it occurs twice under ‘Defense and Security’ where one strategy is given as to “cater for female recruits to ensure the military is able to attract and retain female personnel” [37].

Gender is not mentioned in any of the other eight cross-cutting themes identified, which include, *Youth*, *HIV/AIDS*, *Vulnerable and disadvantaged groups*, *Environment, Climate change*, *Natural disaster management, Public sector management, National statistics systems*. Nor does it occur in any of the other eight parts of the document, which include issues of economic strategies, transport and services development, resource allocations, and implementation and evaluation strategies [37].

In total, “gender” is referred to just 47 times, in just five of the eight documents and together there were just 154 mentions of our searched keywords amongst the total 130,255 words contained in the documents; that is, 0.0012%, or one in every 846 words. Contextually, our reading of the texts suggests that, where gender does occur outside of specific examples of bio-medical treatment, it is considered purely technocratically, either listed along with other areas of concern for equality or as a small disclaimer noting that gender should be considered. Minor exceptions to this general rule were in the *National Research Agenda for HIV and AIDS 2008–2013* and *Papua New Guinea Development Strategic Plan 2010–2030*. Additionally, “gender mainstreaming”, as well as “mainstream” and “mainstreaming” related specifically to gender, appeared in only one document, the *National Research Agenda for HIV and AIDS 2008–2013*.

## Discussion

The first aim of the study was to assess ways in which gender has been represented in PNG’s health sector policy, and to establish the core, and relevant, embedded themes and concepts. Use of the Leximancer tool was intended to scope how gender has been associated with other themes and concepts. However, gendered terms did not manifest sufficiently often in the documents to make adequate assessments. Not only this, but as one of the main criticisms leveled against gender mainstreaming is its technocratic application, simply having a “good” frequency of gendered terms is not enough. The mandate of gender mainstreaming, agreed by the PNG government and aid donors, is that gender be considered at every stage in policy and its application. The traditional researcher-conducted content analysis also showed how the use of gender and gender-mainstreaming terms fell short of comprehensive application, measured both by frequency, and the contextual use, of the terms. All-in-all, gender and gender mainstreaming do not have sufficient prominence in policy documentation to act as a platform for change.

The second aim of our study was to use these results to outline a set of recommendations for strengthening gender equity. PNG is an incredibly diverse country. With one foot in a new modernizing world and the other wedged in its traditions, PNG’s development has been resisted by multiple stakeholders and factors. A lack of political will, despite numerous agreements with donor countries and international treaties signed, means that there has yet to be any genuine commitment made to tackling gender inequality. Beyond this, gender mainstreaming in PNG (and often, elsewhere) does not come with any clear conceptualization of gender. It therefore does not mandate that the complexity of gender be truly considered. In PNG this is particularly relevant as women’s lives intersect with multifarious other factors including their age, education, location and local cultures, health status and economic status.

In the absence of any effective policy platform, our analysis, by extension, permits us to make two recommendations. First, that gender should be re-conceptualized in a way specific to the Papuan context. Second, we argue that emphasis should be placed on those civil society groups which are in a position to make such a relevant conceptualization and which have the capacity, will and desire to effect women’s and men’s lives in PNG. We deal with each in turn.

### Re-conceptualizing gender

The basis for such a re-conceptualization must be to move away from the current biomedical treatment of gender to a more prominent, more inclusive and broad-based, notion. One such lens through which to accomplish this is *intersectionality*. Intersectionality presupposes a way of understanding and analyzing gender as a cross-sectional, multidimensional issue that interplays with all other aspects of people’s identity as well as facilitating an analysis of the structural and political advantages and disadvantages which intersect in women’s lives. It requires that these things be examined together rather than as separate issues [39]. In this way it could provide PNG’s policymakers with a more comprehensive framework for conceptualizing gender, which helps address the diversity of Papuans lives, and work alongside a gender mainstreaming approach.

Gender mainstreaming provides a narrower conceptualization of gender and specific ideas for implementing policies to address the problems it identifies. Conversely, intersectionality offers a more complex, far-reaching framework for a conceptualization of gender and the societal factors that impacts upon it, yet struggles to successfully articulate strategies for effective policy making [39]. However, a synthesis of the two approaches could be a way forward. By combining the strengths of each could result in a more comprehensive policy landscape and a mainstreaming strategy more attuned to the local reality.

### Involving civil society

Having an effective policy environment is only half the story, of course, whether in PNG or elsewhere. The key part of any policy success lies with take-up, adoption and spread; in short with implementation. Civil society groups are hailed as the real change makers in PNG as the feeling pervades that this is where the catalyst for change lies: “The government seems to be dragged along, not leading the way” [11]. Involvement of civil society groups and activists can help firstly, overcome the considerable lack of political will evident in PNG, encouraging citizen participation, awareness and empowerment using non-political approaches to enhance democracy [40, 41]. Secondly, capable civil society groups are often better able to hold their government accountable, ensuring transparency and monitoring policy and effectiveness than other stakeholders [42, 43]. Thirdly, working “on-the-ground”, such groups are better able to create a culturally relevant concept of gender, making it manifest in the lives of people, with more fluid feedback reflecting a changing PNG and the changing status of women. This would be essential to an intersectional approach, and would support any more thoroughgoing attempts at mainstreaming of gender in PNG.

## Conclusion

As Human Rights Watch sadly reports “PNG is one of the most dangerous places in the world to be a woman” [11]. PNG is being hauled into the modern world, and as we have already seen the resistance, and sometimes backlashes to this, are substantial. However, in a well-functioning society, gender cannot be an afterthought. The benefits of gender equity in health policy extend far beyond better healthcare for women. Numerous studies have shown that an improved situation for women is the most effective way to create sustainable long-term development of families and communities, particularly in the developing world [44, 45]. But there is no point in simply mouthing this as some sort of modern day mantra.

In failing to conceptualize gender in PNG effectively or holistically, gender mainstreaming has developed as an approach with little substantial basis, either in policy documentation, or in the reality of women’s and men’s lives [18, 46, 47]. If the aim of gender mainstreaming is to address the socially constructed bases of differences between men and women and to challenge existing gender roles and relations [48], there can be no doubt that it has fallen short of the hopes and aspirations people have had for it, and that in PNG it has thus far failed.

Ultimately, there is no single approach to an issue as complex as gender inequality. Although mainstreaming gender initiatives and promoting intersectionality offer potential as a combined, leveraged way forward, it is necessary to appreciate the broader operating environment that influences organizational priorities. An effective implementation of gender mainstreaming requires both the support of people in decision-making positions and enabling organizations and institutions. We must also be realistic about the impact of policies “on-the-ground”. Stakeholders including aid donors should consider ways to challenge a reticent PNG government, and support civil society groups and activists to raise the profile of gender issues across the board.

## Abbreviations

GDP: Gross Domestic Product
PNG: Papua New Guinea
PNGDSP: Papua New Guinea Development Strategic Plan
